# Corrigendum to “Effect of encapsulation and wall material on the probiotic survival and physicochemical properties of synbiotic chewing gum: study with univariate and multivariate analyses” [Heliyon 5 (7) (July 2019) e02144]

**DOI:** 10.1016/j.heliyon.2019.e02321

**Published:** 2019-08-22

**Authors:** Sahar Dehqan Qaziyani, Amir Pourfarzad, Siamak Gheibi, Leila Roozbeh Nasiraie

**Affiliations:** a Department of Food Science, Nour Branch, Islamic Azad University, Nour, Iran; b Department of Food Science and Technology, Faculty of Agricultural Sciences, University of Guilan, Rasht, Iran

In the original published version of this article, the title and page number of one of the references was incorrectly written. The updated version of the reference is shown below with the changes shown in bold. The authors apologize for this mistake. Both the HTML and PDF versions of the article have been updated to correct these errors.

Najafi, Mohammad B Habibi, Pourfarzad, Amir, Zahedi, Hoda, Ahmadian-Kouchaksaraie, Zahra, & Khodaparast, Mohammad H Haddad. (2016). Development of sourdough **fermented date seed** for improving the quality and shelf life of flat bread: Study with univariate and multivariate analyses. Journal of Food Science and Technology, **53(1), 209–220.**

